# Editorial: Network of pain signaling: from ion channels to brain

**DOI:** 10.3389/fnetp.2023.1297536

**Published:** 2023-11-07

**Authors:** Ekaterina Kutafina, Susanne Becker, Barbara Namer

**Affiliations:** ^1^ Institute of Medical Informatics, Medical Faculty, RWTH Aachen University, Aachen, Germany; ^2^ Clinical Psychology, Department of Experimental Psychology, Heinrich Heine University, Düsseldorf, Germany; ^3^ Integrative Spinal Research, Department of Chiropractic Medicine, University Hospital Balgrist, University of Zurich, Zurich, Switzerland; ^4^ Department of Cognitive and Clinical Neuroscience, Central Institute of Mental Health, Medical Faculty Mannheim, Heidelberg University, Mannheim, Germany; ^5^ Research Group Neuroscience, Interdisciplinary Center for Clinical Research Within the Faculty of Medicine, RWTH Aachen University, Aachen, Germany; ^6^ Department for Neurophysiology, Medical Faculty, RWTH Aachen University, Aachen, Germany

**Keywords:** pain, nociception, networks, multi-scale, interdisciplinary, transdisciplinary

The complexity of the percept “pain” is remarkable, even in the context of medical and neuroscientific research. There are many factors, from biological to psychological, involved in the modulation of how a nociceptive signal is processed. Due to this variability, pain perception can be considered a reflection of a state of an interactive and dynamic network, in which large amount of information from various sources is integrated. Within such a network, we can, for example, zoom on molecular structures of ion channels, consider cellular levels, or pain-linked genetic mutations. Zooming out, integration of processes of the central nervous system, including effects of psychological factors, is an important part of such network. Handling this level of complexity satisfyingly requires interdisciplinary research and communication. Speaking in terms of networks, it is important to continue pushing individual nodes of knowledge, i.e., specialized fields of research within a domain, forwards, but also build strong network “links,” i.e., connections between these nodes, starting from closely related nodes and progressing towards increasing complexity of the covered network patches. Such a network construction should go even beyond the pain research community: there are strong links to other medical domains, such as neurogenerative disorders, diabetes, and autoimmune diseases.

In this Research Topic “*Network of Pain Signaling: From Ion Channels to Brain*” we aimed to collect some ideas how the network physiology approach can be applied in pain research.

With the aim to support knowledge transfer in interdisciplinary projects involving computational researchers without medical or neuroscientific background and to set up a basic framework for the idea of constructing pain models from a network perspective we provided a review. Every person getting involved in pain research asks at one point the obvious question: “How to measure pain?”. Although it appears to be a simple question, it targets “the elephant in the room.” Because of its subjectivity, pain is notoriously hard to assess. To understand this issue, we emphasize the difference between pain as a conscious and multidimensional percept and nociception as a signaling process. Accordingly, we present and discuss different approaches and modalities of research linked to the assessments of objective indicators of nociceptive processing as well as the assessments of the subjective experience of pain. Importantly, we aimed to describe this information in a manner also suitable for non-experts. With this review, we would like to encourage our colleagues to contribute to interdisciplinary knowledge transfer, by writing summaries and reviews suitable for boosting collaborative work.

A great illustration of the necessity and potential of interdisciplinary research is shown in the paper of Häfele et al.. It combines molecular research on ion channel level with fMRI of the brain in rodents, rendering it an example of how to integrate outcome measures of very different processing levels. This work illustrates the importance of animal models in studies on pain mechanisms, highlighting fine-grained insights impossible to get in human studies. Moreover, the population of genetically modified rodents was compared here to a control group through assessment of a rich panel of modalities including sensitivity tests, behavioral responses, and fMRI. The informational integration of these data illustrates very well a “network cluster” in pain research. Further, the fMRI data itself is analyzed for network properties and with that the work highlights the fractal structure of the pain network landscape.

Focusing on prevalent issues in the translation from preclinical to clinical trials specifically related to the assessment of pain, Jonas and Schmelz provide an insightful perspective article. The perceptual nature of pain implies a necessary choice between assessment of nociceptive processes (typically not reflecting the pain experience), and assessment of the subjective experience through self-assessment. Importantly, the choice of methods that can be used in both animal and human subjects overlaps only limitedly, hampering the success of translational research. Specifically, the authors highlight conceptual difficulties when using threshold testing as psychophysical output for ongoing spontaneous pain and suprathreshold pain. In sum, they advocate for integrative measurements, in which multiple assessments are optimized as a clinically relevant quantifier.

The fourth manuscript by Yang et al. adds insights in the use and value of network-based analyses of brain data, specifically morphometric features of the brain derived from multimodal magnetic resonance imaging (MRI) data. Using a sophisticated analysis method to describe morphometric similarity networks, the authors show that these networks can discriminate between patients with lumbar disc herniation and chronic pain, and healthy controls. Although such morphometric networks are not specific to pain, they are able to predict statistically to some extend clinical pain reported by the patients. These findings add an interesting dimension to the network perspective in pain, illustrating how such unspecific morphological alterations can be linked to the experience of pain. Thus, these insights further emphasize the added value of a network approach over the use of single or separate measurements and can be viewed as an example of successful translation to clinically relevant assessments.

In sum, this Research Topic is one of the first steps to embed pain research in a framework of network physiology and voice the necessity of community discussions about methods and strategies for developing the multi-modal, multi-scale network models. Efficient knowledge transfer and interdisciplinary communication are cornerstones of successful research, in particular when aiming to translate important fine-grained insights to clinically relevant aspects of pain assessment and, in the long run, also to efficient treatment of chronic pain.

